# Analysis of Rock Varnish from the Mojave Desert by Handheld Laser-Induced Breakdown Spectroscopy

**DOI:** 10.3390/molecules26175200

**Published:** 2021-08-27

**Authors:** Russell S. Harmon, Daria Khashchevskaya, Michelle Morency, Lewis A. Owen, Morgan Jennings, Jeffrey R. Knott, Jason M. Dortch

**Affiliations:** 1 Department of Marine, Earth, and Atmospheric Sciences, North Carolina State University, Raleigh, NC 27695, USA; dkhashc@ncsu.edu (D.K.); mjmoren2@ncsu.edu (M.M.); lewis.owen@ncsu.edu (L.A.O.); 2 SciAps, Inc., 7 Constitution Way, Woburn, MA 01801, USA; mjennings@sciaps.com; 3 Department of Geological Sciences, California State University, Fullerton, Fullerton, CA 92831, USA; jknott@fullerton.edu; 4 Kentucky Geological Survey, University of Kentucky, Lexington, KY 40508, USA; Jason.M.Dortch@uky.edu

**Keywords:** laser-induced breakdown spectroscopy (LIBS), rock varnish, geochemical fingerprinting, microchemical mapping, desert pavement

## Abstract

Laser-induced breakdown spectroscopy (LIBS) is a form of optical emission spectroscopy that can be used for the rapid analysis of geological materials in the field under ambient environmental conditions. We describe here the innovative use of handheld LIBS for the in situ analysis of rock varnish. This thinly laminated and compositionally complex veneer forms slowly over time on rock surfaces in dryland regions and is particularly abundant across the Mojave Desert climatic region of east-central California (USA). Following the depth profiling examination of a varnished clast from colluvial gravel in Death Valley in the laboratory, our in situ analysis of rock varnish and visually similar coatings on rock surfaces was undertaken in the Owens and Deep Spring valleys in two contexts, element detection/identification and microchemical mapping. Emission peaks were recognized in the LIBS spectra for the nine elements most abundant in rock varnish—Mn, Fe, Si, Al, Na, Mg, K, Ca and Ba, as well as for H, Li, C, O, Ti, V, Sr and Rb. Focused follow-up laboratory and field studies will help understand rock varnish formation and its utility for weathering and chronological studies.

## 1. Introduction

Rock varnish is a globally common geomorphic feature that slowly accumulates over time on rock surfaces in dryland regions. This dark-colored, finely-laminated veneer is composed of poorly crystallized Fe–Mn oxyhydroxides contained in a matrix of detrital clay minerals and silica. Varying in color from shades of orange, mahogany, brown and black, rock varnish characteristically comprises a tens to hundreds of micrometers thick cyclic accretionary sequence of discontinuous dark and light micron-thick layers of variable morphology, mineralogy and chemistry [1,2,3,4,5,6,7]. Understanding the origin of rock varnish has recently become a subject of particular research interest [8,9,10,11,12,13,14,15,16] because of its potential as a paleoclimate proxy [17,18,19,20] and as a terrestrial analog to rock surface coatings on Mars [21,22,23,24]. Although a variety of microanalytical techniques have been used to examine rock varnish in the laboratory, in situ examination of rock varnish is challenging. Here, we present an innovative approach for rapid elemental analysis of rock varnish in the field using handheld laser-induced breakdown spectroscopy (LIBS).

LIBS is a simple and robust analytical technique for chemical analysis in which a high-energy laser beam is tightly focused onto the surface of a sample to ablate a small amount of material into high-temperature plasma. The plasma light is collected and analyzed to determine elemental composition of the sample. LIBS can be used in five distinct ways: (1) for elemental profiling and identification; (2) for depth profiling of a sample; (3) for 2D or 3D microchemical mapping; (4) for element abundance quantification; (5) for distinguishing between materials of similar composition through chemometric analysis of LIBS data. For this study, we used a commercial handheld LIBS instrument for both laboratory and in-field analysis of rock varnish from the Mojave Desert climatic region of east-central California (USA).

## 2. Chemical Analysis of Rock Varnish

Chemical analysis over the past half-century by a variety of analytical techniques has shown that rock varnish is particularly rich in Mn, Fe and Ba, with its brown–black layers rich in Mn and yellow–orange layers rich in Si and Al but depleted in Mn [1,3,8,9,10,15,17,18,19,20,23,24,25,26,27,28,29,30,31,32,33,34,35,36,37,38,39]. These studies have documented the abundance of four elements (Al, Si, Mn and Fe) at concentrations of >10 wt% and of another seven elements (Na, Mg, P, K, Ca, Ti and Ba)—at concentrations of >0.1 wt%. The enrichment of certain minor and trace elements (Co, Ni, Cu, the REE, Pb, Th and U) is attributed to a combination of photooxidation of Mn under intense solar radiation and subsequent oxyhydroxide scavenging in an aqueous environment [8,14,15,35]. Mn concentrations within varnish layers, typically between 10–30 wt%, are similar over large geographic regions, but rock varnishes from different geographic regions are distinguishable based on different trace element profiles and variable elemental ratios [10,15,36]. Compositional variation in Mn is thought to be mediated by variations in environmental moisture [8,34] through episodic cycles of surficial wetting and drying that occur at diurnal, annual and millennial times scales, with Mn concentrations elevated during episodes of wetter climate [8,17,18,19,20].

Spectroscopic analysis has revealed that aluminosilicate clay minerals are the major constituent of rock varnish [1,2], with Mn–Fe oxyhydroxides dispersed throughout this matrix. Subsequent work [13,22,37,40] determined that the Mn component is a mixture of a birnessite-like phase [(Mn^4^⁺,Mn^3^⁺)_2_O₄·1.5H_2_O] rich in Ba and Ca, buserite [Na_4_Mn_14_O_27_·21H_2_O] and a mineral of the hollandite (BaMn^4^⁺_6_Fe^3^⁺_2_O_16_)–todorokite [(Na,Ca,K,Ba,Sr)_1−__X_(Mn,Mg,Al)_6_O_12_·3–4H_2_O] group. Domains of amorphous hydrated silica have been documented in rock varnish using Raman spectroscopy and scanning transmission electron spectroscopy [41]. Rock varnish is generally agreed to be an authigenic surface coating, with its aluminosilicate, oxyhydroxide and silica components derived from dust that has settled into microdepressions on rock surfaces through atmospheric deposition [1,8,15,29,35,38,40,42], an inference supported by the presence of short-lived isotopes ^137^Cs and excess ^210^Pb in the surface layer rock varnish [43,44].

Biological mediation by chemolithotrophic bacteria that precipitate Mn from a solution by oxidizing Mn^2+^ and Mn^3+^ to Mn^4+^ may play a role in Mn fixation and concentration [45,46,47,48,49,50,51,52], but some have argued that Mn enrichment in rock varnish is largely an abiotic inorganic process involving leaching, mobilization and photochemical oxidation [8,12,14,15,35] and that silicic acid acts as a mechanism for elemental mobilization, complexation and cementation [41]. Recently, Lingappa et al. [16] described a unifying ecophysical model for rock varnish formation. Their model explains rock varnish as an aerobic microbial ecosystem governed by sunlight, water and manganese redox cycling operating in the moist environment of microdepressions on rock surfaces where dust accumulates, and photosynthetic cyanobacteria sequester high concentrations of Mn in their cells and exploit the unique redox chemistry of manganese complexes as a catalytic antioxidant system. The cyanobacterial post-mortem biomass residue provides then the high-Mn reservoir that is subsequently oxidized, mobilized and redistributed to form the oxyhydroxide and silica cement of rock varnish.

## 3. Laser-Induced Breakdown Spectroscopy

### 3.1. Background

LIBS is a straightforward form of atomic emission spectroscopy for rapid and in situ multielement analysis of a material in any physical state—solid, liquid or gas [53]. LIBS instrumentation is both simple and robust. A LIBS analytical system consists of only a few components: (1) a solid-state short-pulsed Q-switched laser used to create microplasma on the sample; (2) optics to first focus the laser light onto the sample and then collect the light emitted from the plasma; (3) a coupled fiber optic and spectrometer/detector system for acquisition and spectral resolution of the optical emission; (4) a computer for system control and data processing. LIBS has sequentially advanced from bespoke to commercial laboratory systems and, most recently, to commercial handheld analyzers [54].

In LIBS, high-energy laser light of short pulse duration sequentially ablates, vaporizes and dissociates a small volume of a sample to form high-temperature plasma comprising a mixture of free electrons and weakly ionized and ionic, atomic and molecular species. Plasma cooling causes species recombination and de-excitation, with light emission occurring at discrete wavelengths when electrons return to lower energy levels. The character of the microplasma created during LIBS analysis of a solid sample is complex and determined by multiple interrelated factors that include the operational characteristics of the laser (i.e., wavelength, energy and pulse duration), the nature of the material being ablated (e.g., material type, composition, crystallinity, optical reflectivity and surface texture), the degree of laser energy coupling to the sample surface and the ambient environment in which the LIBS plasma is formed [53,55].

Sample chemical composition can be determined spectroscopically by monitoring the position and intensity of emission lines in the LIBS emission spectrum. Since spectral emission intensity is proportional to the abundance of an element present in a sample, quantitative analysis by LIBS is possible but requires closely matrix-matched standards. LIBS elemental detection limits are highly variable, but can be in the low tens of the ppm range for many elements. Elements on the left side of the periodic table (alkali metals and alkali earths), such as Li, Na and Ba, that have low ionization energy typically display strong emission and, therefore, can be detected at very small abundances. By contrast, non-metallic elements on the right side of the periodic table with high ionization potential, such as halogen elements, are more challenging for determination by LIBS and consequently have much higher limits of detection. In complex matrices, transition metals typically display numerous low-intensity spectral lines.

LIBS can provide multielement detection and quantification in real time because all elements have optical emission lines over the 200–900 nm spectral range so that a single laser shot provides a broadband optical emission spectrum for a sample that records its complete chemical composition (i.e., a “geochemical fingerprint”). Since many electron orbital transitions occur for most elements, a LIBS broadband spectrum typically consists of multiple peaks for most elements and contains tens to hundreds of spectral lines for most geological materials. Handheld LIBS is especially well-suited to geochemical applications that would benefit from real-time analysis outside the traditional laboratory setting and during geologic fieldwork because of its portability and versatility [56,57]. When used in conjunction with statistical chemometric data processing techniques, LIBS allows for the discrimination of samples of similar appearance but different composition, stratigraphic correlation or the determination of geomaterial provenance [58].

In addition, LIBS sampling is highly resolved spatially as the plasma forms over a restricted spatial area of tens of micrometers on the sample surface, with only a minute quantity of material (typically picograms to nanograms) ablated and sampled by each laser pulse. This precise resolution permits in situ analysis of individual particles, mineral grains or inclusions; the fine-scale compositional mapping of compositionally or texturally complex samples; the analysis of thin crusts, coatings or surface alteration zones without substrate interference; chemical analysis at highly spatially resolved spatial scales < 10 μm [58]. Stratigraphic analysis of a sample by depth profiling is also possible as sequential ablation with successive laser pulses progressively bores down into a sample.

### 3.2. Previous LIBS Analysis of Rock Varnish

**Figure 1:**
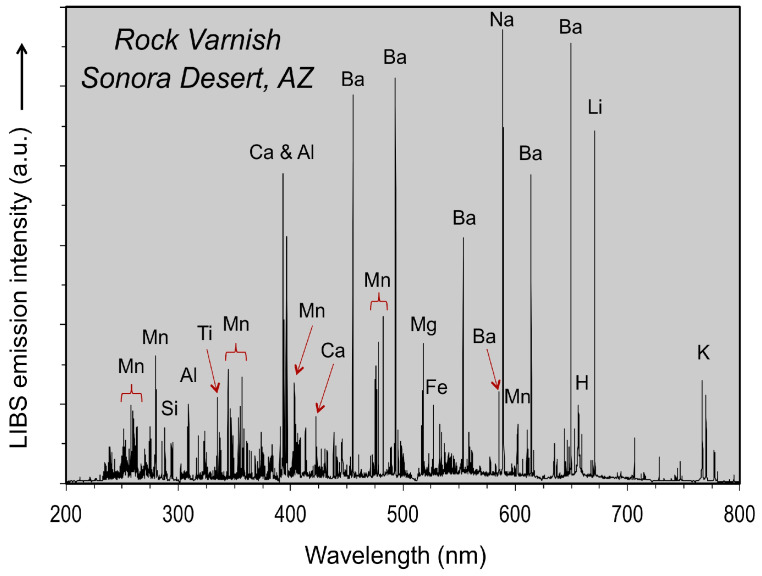
LIBS broadband spectrum acquired with a commercial laboratory LIBS system (Ocean Optics, Inc., Dunedin, FL, USA) for the surface of a varnished quartzite cobble from the desert pavement developed on an alluvial fan in the Sonora Desert near Yuma, Arizona.

It has been demonstrated in the laboratory that LIBS analysis can be used to determine the elemental composition of rock varnish on a quartzite cobble from the desert pavement in the Sonora Desert of southwestern Arizona (USA) and successively bore into the specimen to examine elemental variation stratigraphically through the varnish layer [59]. Emission lines were observed for Mn and Fe, Si and Al, H and the alkali and alkali earth elements Li, Na, Mg, K, Ca and Ba (Figure 1).

It is important to note that relative emission line intensities in Figure 1 are not indicative of absolute elemental abundances, but instead reflect a combination of element emissivity and concentration. Thus, the strong intensity for the Li, Na, Ca and Ba spectral lines is the direct consequence of the low ionization potential of these elements, despite the modest concentrations expected to be present within the varnish [10,15,31,33,36]. The intensity of the Mn and Ba lines, which are most pronounced in the surface layer formed under present-day climate conditions, progressively decreased in intensity relative to the Si peak in subsequent laser shots. Similarly, the peaks for the alkali and alkali earth elements decreased steadily into the varnish layer from the surface.

Two subsequent studies conducted in the laboratory [24,25] used a vacuum chamber at low pressure and standoff LIBS analysis at a distance of up to 5 m to analyze varnish coatings on basalts from the Black Point lava flow in the San Francisco Volcanic Field near Flagstaff, Arizona (USA). This work, undertaken in the context of extraterrestrial LIBS application by the ChemCam system on the Curiosity rover on Mars, demonstrated that both a bespoke laboratory LIBS system and an engineering model of the ChemCam LIBS system were successful for standoff analysis at the 3–5 m distance. For the latter LIBS system, which had a laser spot size of ~400 μm, the LIBS spectra acquired over limited ranges of the ultraviolet (240–342 nm), violet (382–469 nm) and near-infrared (470–907 nm) spectral regions documented that the thin (<1 mm) dark varnish layer had an overall composition different from its basalt substrate, containing H, Li, Na, Mg, Al, Si, K, Ca, Ti, Mn, Fe and Ba. Depth profiling with 300 laser shots to ~90 μm depth observed that Mn, Ba and H were high in the surface region and then exhibited a sharp decrease into the varnish layer.

## 4. Analytical Methodology

### 4.1. Z-300 Handheld LIBS Analyzer

The work reported here utilized a Z-300 (SciAps, Inc., Woburn, MA, USA) LIBS analyzer, a handheld instrument with dimensions of ~21 × 29 × 11 cm that weighs 1.8 kg and is powered by an onboard Li-ion battery that during field use typically provides ~500 complete analyses. This instrument employs a Class 3 PULSAR^TM^ 1064 nm Nd:YAG diode-pumped solid-state pulsed laser producing a 100 μm focused beam that delivers a 5–6 mJ pulse to the sample at the 1 ns pulse duration for a firing rate between 1 and 50 Hz. As the laser is hazardous for eye exposure, the Z-300 analyzer contains built-in safety interlocks which prevent the laser from firing when no sample is present in front of the laser aperture. This allows the analyzer to be operable under Class 1 conditions so that no protective eyewear is required. The analyzer can operate in ambient atmosphere, but also is capable of gas purging that delivers an inert gas (usually Ar) from a canister in the instrument handle directly to the focusing area on the sample surface where LIBS plasma formation occurs, thus producing plasma containment and emission signal enhancement.

Z-300 records a broad range of plasma light emission, from 190 to 950 nm, a spectral range over which every element has at least one emission line. The LIBS emission light signal is collected and passed via a fiber optic cable into three internal spectrometers utilizing time-gated CCD detectors with respective spectral ranges and resolutions of 190–365 nm with FWHM of 0.18 nm, 365–620 nm with FWHM of 0.24 nm and 620–950 nm with FWHM of 0.35 nm. Spectral data are typically collected with a 650-nanosecond delay time over the 1-millisecond integration time. However, the detector can be operated in a non-gated state or with variable gate delays ranging from 250 to 10,000 nanoseconds.

Regular wavelength calibration of the spectrometers is performed by interrogation of an internal target made from Grade 316 Mo-bearing stainless steel. For each spectrometer in the unit, wavelength errors between the selected observed emission lines and the values found in the National Institute of Standards and Technology (NIST) Atomic Spectra Database [60] are determined and correction coefficients are applied. These new coefficients are used until the next wavelength calibration is performed, and an onboard record of all calibration spectra and correction values is kept.

The analyzer also has a video camera for viewing the sample before analysis and a laser spot finder to show where the laser beam will strike the sample. It also contains an onboard 3D translational stage that is computer-controlled for automated laser focus at the sample location and rastering of the laser beam across the sample in the XY-direction at discrete locations for targeted analysis or data averaging. The raster pattern is acquired at 12.5 μm steps over an area of up to 2 × 2 mm^2^, with the size of the grid and the number of laser shots fired at each raster location defined by the user. Furthermore, if desired, a user-selected number of non-analytical shots can be performed for surface “cleaning” prior to the collection of spectral data. Typical detection limits are in the tens to hundreds of parts per million of the ppm range for most elements when averaged across the 2 mm^2^ area of analysis.

### 4.2. Data Processing Software

Z-300 has an Android operating system with a graphic user interface (GUI). The spectral data acquired are stored in the instrument and then accessed via the GUI downloaded to a computer communicating with the analyzer via USB or Wi-Fi or emailed using a mail account set up on the instrument when connected to Wi-Fi. The analyzer has three onboard applications for data processing and analysis: Element Pro, GeoChem and GeoChem Pro. The Element Pro mode is used for rapid, qualitative analysis. Each acquired spectrum is searched using a proprietary onboard spectral library of LIBS emission lines and relative emission strengths for the entire periodic table. A list of the elements identified in the sample is displayed after each analysis, which is accompanied by a “likelihood” rating and an estimated elemental “relative abundance”. The former is a measure of the ratio of the number of elemental emission lines present in an acquired spectrum to the number of lines for each element in the spectral library. The latter estimates how much of an element is present in the sample compared to other elements with the caveat that there is no direct correlation between relative abundance and absolute element concentration.

The GeoChem mode is used for quantitative analysis based upon a previously developed empirical calibration curve, whereas the GeoChem Pro mode for qualitative analysis can identify the spectral peaks of specific elements and generate relative concentration maps based upon the recorded intensities of selected elemental peaks across a raster pattern. Complete spectral data are collected and analyzed at each point of the raster pattern. The ablation crater formed from a single laser shot is 50–100 μm, depending on material type, so that a typical raster map displays variations in elemental abundance across a 16 × 16 grid pattern over the 2 × 2 mm area. Spectral signatures at each grid point are compared to the onboard library to estimate elemental abundances. Acquired raster patterns are displayed as “heat maps” for any element of interest present in the sample; areas of high concentration are displayed in red, of low concentration—in blue, with a gradient of colors for intermediate concentrations. These heat maps are saved as JPG files that can be exported for post-analysis processing with third-party software [57].

### 4.3. Data Collection

Our LIBS analyses were undertaken in three ways. First, averages of four recorded spectra were collected after two cleaning shots at the laser-firing rate of 50 Hz over a 4 × 3 grid to be used for elemental identification using the Element Pro software. Then, microscale mapping was undertaken with a single laser shot at the laser-firing rate of 10 Hz at 256 locations over a 16 × 16 grid to obtain elemental distribution heat maps. The data file for each laser shot is a .csv file containing the wavelength and the relative intensity value (i.e., the number of photons counted) for each of the 23,432 channels of the spectrometer.

## 5. Results and Discussion

LIBS spectra, similar in form to those shown in Figure 1, were obtained using two Z-300 commercial handheld LIBS analyzers. Before going into the field, one handheld analyzer was used to examine Death Valley sample JRK-DV-72 in the laboratory. Subsequently, another instrument was used in the field for in situ LIBS analysis of varnished basalt lava and varnished rocks on desert pavements covering alluvial fans as well as visually similar fracture fillings on granite and surface coatings on granite outcrops and boulders.

### 5.1. Laboratory Analysis

**Figure 2:**
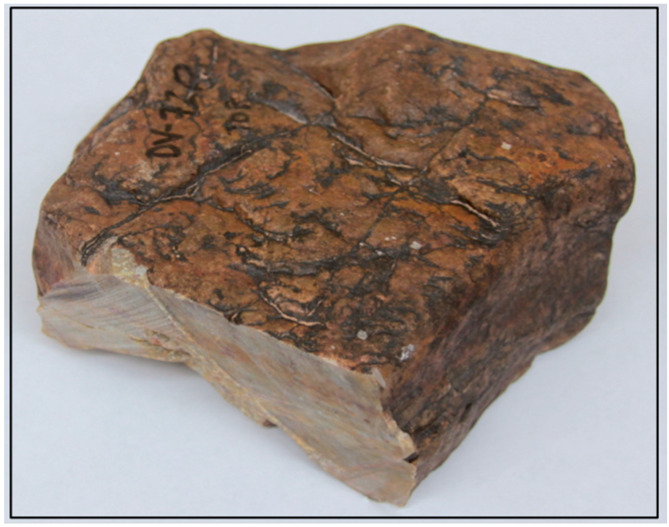
Sample JRK-DV-72, a strongly varnished clast of quartzite from Death Valley, California (36.38513 N, 118.84396 W).

**Figure 3:**
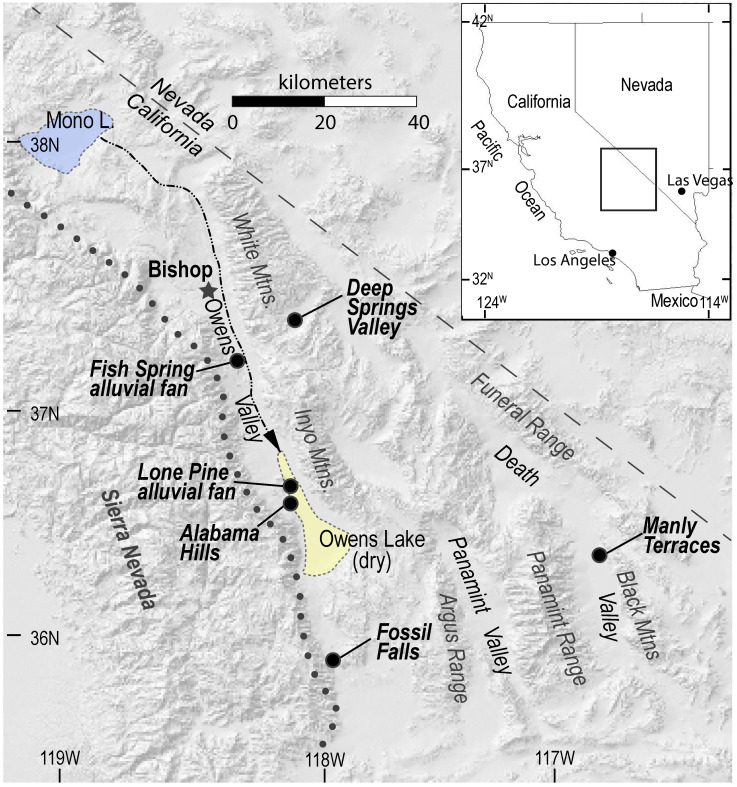
Shaded-relief map of eastern California and western Nevada showing the Manly Terraces, Fossil Falls, Alabama Hills, Lone Pine alluvial fan, Fish Springs alluvial fan and Deep Springs Valley sites sampled during this study. The western boundary of the Mojave Desert is indicated by the dotted line. The Owens River (black dashed line) flows in the southeasterly direction from Mono Lake (blue) to Owens Lake (yellow).

Before our Mojave Desert fieldwork, the varnished surface of sample JRK-DV-72 and its substrate were analyzed in the laboratory. This sample (Figure 2) is a clast of Ordovician Eureka Quartzite from colluvial gravels deposited as the Manly Terraces (Figure 3) of Pleistocene Lake Manly in Death Valley, California [61]. A cosmogenic ^10^Be exposure age of ~109 Ka has been determined for the Manly Terraces [62]. Sample JRK-DV-72 has a strongly varnished top surface (Munsell color = 5 R 2/2 to 5 R 4/2) and Fe-stained base (Munsell color = 10 R 4/6 to 10 R 6/6) that was in contact with the underlying soil upon which it rested.

### 5.2. Field Analysis

**Figure 4:**
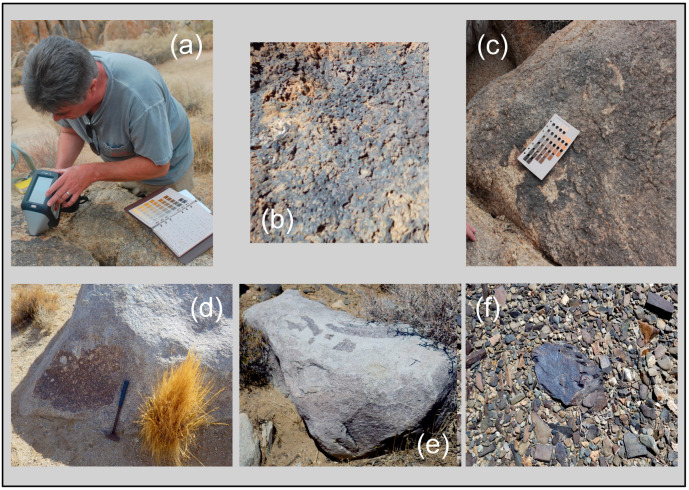
(**a**) Typical use of handheld LIBS during fieldwork. (**b**) Discontinuous varnish on basalt at Fossil Falls, Owens Valley. (**c**) Desert varnish on a granite outcrop, Alabama Hills, Owens Valley. Residual desert varnish “patches” on granite boulders on the (**d**) Lone Pine and (**e**) Fish Springs alluvial fans of Owens Valley. (**f**) Strongly varnished clasts in the desert pavement on an alluvial terrace in Deep Springs Valley.

**Figure 5:**
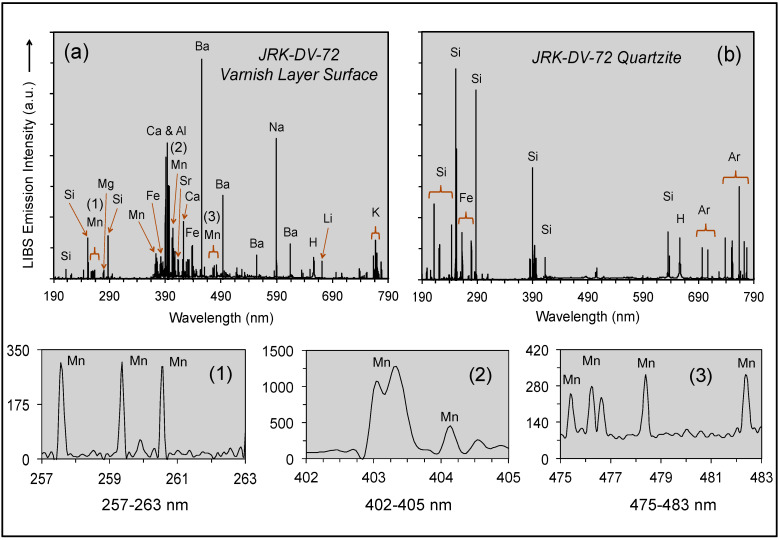
LIBS spectra acquired in the laboratory for (**a**) the varnish layer on the top surface of sample JRK-DV-72 and (**b**) its quartzite substrate.

**Figure 6:**
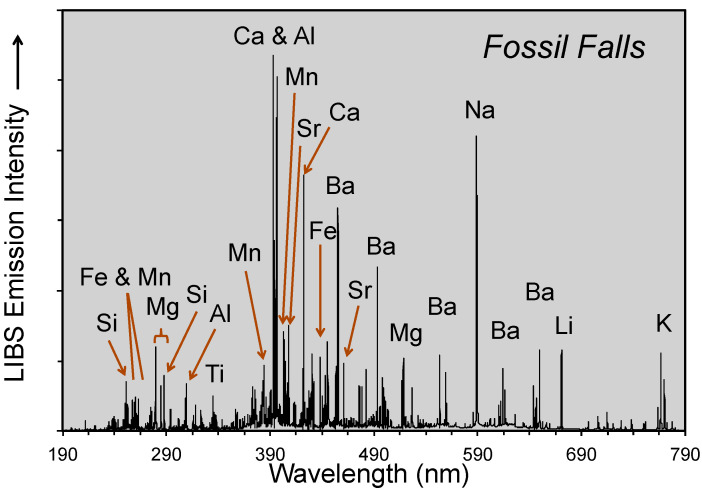
Z-300 LIBS spectra for rock varnish at the Fossil Falls locality on basalt lava at the Fish Springs locality.

**Figure 7:**
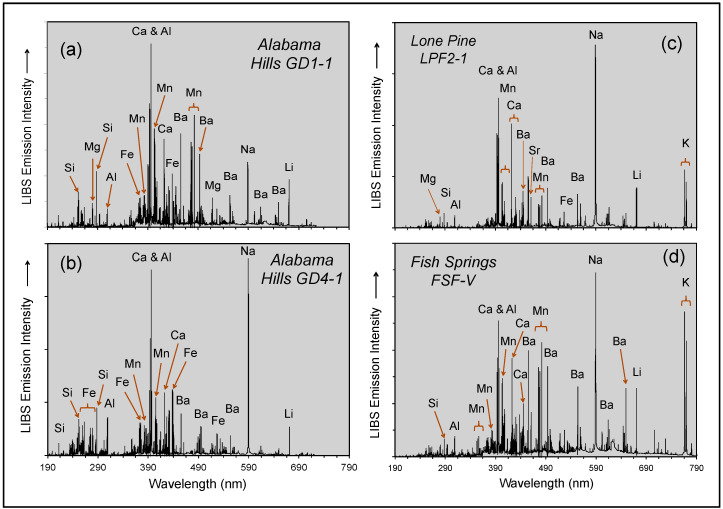
LIBS spectra for rock varnish in fractures and fissures in highly weathered monzogranite at the Alabama Hills locality (**a**,**b**) and on large granitic boulders residing on the Lone Pine (**c**) and Fish Springs (**d**) alluvial fans.

**Figure 8:**
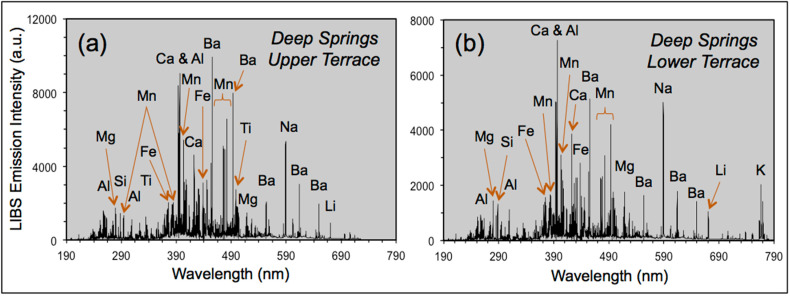
LIBS spectra for varnished clasts on the desert pavement covering alluvial fans on the upper and lower terraces in Deep Springs Valley.

LIBS analytical work was undertaken at five localities across the Mojave Desert (Figure 3) during our geologic fieldwork in June 2021. The use of the LIBS handheld analyzer in the field and examples of field occurrences analyzed are shown in Figure 4, with representative LIBS spectra shown in Figure 5, Figure 6, Figure 7 and Figure 8. Five sample types were analyzed:

- Surface rock varnish on basalt at Fossil Falls in the southern Owens Valley;
- Surface rock varnish on quartzite clasts from desert pavements covering two alluvial fans of different ages in Deep Springs Valley;
- Surface coatings on granite at the Alabama Hills in Owens Valley;
- Fracture fillings along joints in granite at the Alabama Hills;
- Surface coating on granitic boulders on the Lone Pine and Fish Springs alluvial fans in Owens Valley.

Fossil Falls, located ~30 km south of Owens Lake, is where the vesicular basalt of the Red Hill lava flow [63] filled the valley floor and dammed the pluvial Owens River. The Owens River eventually overtopped the flow, with water from the lake spilling across the basalt sill during nine high lake-level stands from ~40 to 10.6 ka [64]. The spillway has impressive water-worn lava with meter-deep potholes and a paleo-waterfall that formed a >20 m deep gorge. One sample of varnish developed on basalt at this locality was analyzed, FF-1 (35.970367 N, 117.908367 W; Munsell color = 10 YR 2/2, dusky yellowish brown).

The Alabama Hills are a downfaulted block of Late Cretaceous porphyritic monzogranite [65,66,67]. This pervasively jointed and deeply weathered granite forms inselbergs, tors and pediments that have weathered to an orange–brown color and display a well-developed and multicolored coating of post-depositional origin in their outcrop area [68]. Two such samples from the Alabama Hills were analyzed: DG1-1, multicolored varnish on a joint surface on a strongly fractured outcrop (Munsell color = 7.5 YR 3/1, very dark gray), and GD4-1, a thick layer on a large boulder (36.585262 N, 118.11523 W; Munsell color = 10 YR 2/1, black).

Juxtaposed with the Alabama Hills are Quaternary boulder-bearing debris flow deposits and alluvial fans forming bajadas that extend from the Sierra Nevada mountain front through the Alabama Hills to the lakebed floor of Owens Valley [66,69]. Cosmogenic exposure ages imply that deposition on three alluvial fans at the Lone Pine Creek locality ceased at 21, 8 and 1 ka, respectively [70]. Later work [69] confirmed the ages of these alluvial fans by additional measurement of ^10^Be, and the fans were reinterpreted to be the result of glacial lake outburst floods. One of the samples analyzed at this locality, LPF2-1 (36.607082 N, 118.075762 W; Munsell color = 7.5 YR 3/0, very dark gray), is a dark patch on an isolated granodiorite boulder with the dimensions of ~6 × 4 × 2.5 m on the middle part of the alluvial fan (Figure 4d). This coating on the granite boulders here and on the boulders on the Fish Springs alluvial fan are residual patches remaining after abrasion of the boulders during glacial and debris flow transport and the physical weathering after range fires that caused spallation through uneven heating [71].

The Fish Springs alluvial fan is an older debris flow with weathered granitoid clasts on the northern and western sides of the Fish Springs cinder cone [68]. One of the samples analyzed from this locality, FSF-V (37.08225 N, 118.269483 W; Munsell color = 10 YR 4/1, dark gray), is a small dark patch on a flat-topped, fine-grained granite boulder with the dimensions of ~3 × 2 × 1.5 m characterized by small weathering pits across its upper surface (Figure 4e).

The samples analyzed from Deep Springs Valley are from desert pavements formed on Pleistocene alluvial fan deposits (Qoa of [72]). The drainage basin source for these deposits is underlain by Cambrian bedrock units of a wide variety of lithologies, including quartzitic sandstones. Based on our observations, we subdivide the older alluvial fan into an older fan and an intermediate-age fans. Both fan units are covered by a well-developed desert pavement and vesicular A soil horizon. The surface of the older fan is ~10 m above the active channel, whereas the intermediate fan is higher, at ~6 m above the active channel. Mapped shorelines from the Pleistocene Deep Springs lake are present only on the older alluvial fan [72]. Based on regional geomorphology, both the older and intermediate-age fans are considered to be younger than 60 ka [73,74]. Sample T5 is from the upper terrace (37.31706 N, 118.090472 W; Munsell color = 5 YR 3/1, very dark gray), sample T13—from the lower terrace (37.31656 N, 118.089933 W; Munsell color = 10 YR 3/1, very dark gray). These desert pavements are strongly varnished and display the wide variety of colors characteristic of rock varnish across the Mojave Desert (Figure 4f).

### 5.3. LIBS Elemental Detection and Identification

Examples of broadband LIBS spectra from our analysis of rock varnish and coatings are shown in Figure 5, Figure 6, Figure 7 and Figure 8. These are unprocessed spectra that have not been baseline-corrected or normalized. The differences in the form of the spectra shown in these figures represent a combination of the extent of laser energy coupling to the samples and their variations in elemental abundances.

As noted above, specimen JRK-DV-72 is a strongly varnished clast of Ordovician Eureka Quartzite from colluvial gravels deposited on the Manly Terraces of Pleistocene Lake Manly in Death Valley, California (36.38513 N, 118.84396 W; 5 R 2/2 to 5 R 4/2). The LIBS spectrum of the varnished surface of this sample, obtained in the laboratory before our fieldwork, is presented in Figure 5a. The strong compositional contrast between the rock varnish and its quartzite substrate is apparent by comparison with Figure 5b. Since Mn is an element of particular importance in rock varnish, but also an element characterized by weak spectral emission lines spread over multiple parts of the 190–790 nm spectral range shown in Figure 5a, documenting its presence in the LIBS spectra is important. Thus, three specific spectral regions of interest for Mn (257–263, 402–405 and 475–483 nm) are also shown in panels (1), (2) and (3) of Figure 5. The broad peak at 403 nm is a composite of the three most intense peaks for Mn, closely spaced at 403.08, 403.37 and 403.45 nm. Another Mn triplet similar in form to those of panels (1) and (3) is present between 293 and 296 nm but is not shown in the figure.

As noted in Table 1, five studies [9,15,35,36,39] analyzed rock varnish from the arid regions of western Nevada and California quantitatively using inductively coupled plasma mass spectrometry (ICP-MS). These studies documented the presence of 55 elements—10 major elements with abundances exceeding 1 wt% and 45 minor, trace and rare earth elements with concentrations at levels between tenths to thousands of ppm. Mn, Fe, Si and Al are the predominant elemental constituents of rock varnish across the region.

Our analysis of rock varnish in the Mojave Desert of California by handheld LIBS recorded the presence in the spectra between 200–900 nm of more than 300 emission lines for 17 elements (Table 1 and Table 2). This includes the 10 elements documented in Table 1 as the most abundant in rock varnish: Mn and Fe; Si and Al; alkali elements Li, Na and K; alkali earth elements Mg, Ca and Ba. In addition to the 45 spectral peaks for these elements, other peaks of low intensity are recognized for the primary spectral lines of Ti, V, Sr and Rb. One of the premier features of LIBS is its capability of analysis of elements of low atomic weight, hence our recognition of H, Li, C and O emission lines in the handheld LIBS spectra. The broad characteristic H_α_ Balmer peak at 656.3 nm, which is considered diagnostic of the presence of water in geologic materials [75], is strong in Death Valley sample JRK-DV-72 (Figure 5), but also is present in the LIBS spectra of all Owens Valley and Deep Spring Valley samples. The presence of Li, which in the environmental context of Owens Valley has been introduced into the local atmosphere as dust generated from lake bed sediments during interpluvial climatic episodes, illustrates the propensity of this highly emissive element to appear in LIBS spectra wherever present in a local geologic environment even in low abundance.

#### 5.3.1. Microscale Mapping

**Figure 9:**
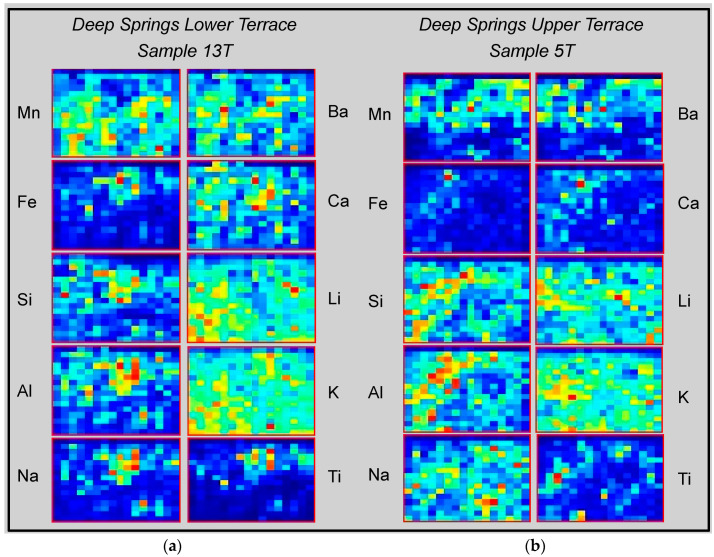
Elemental heat maps obtained by Z-300 raster scanning of strongly varnished desert pavement clasts on the lower (**a**) and upper (**b**) terraces in Deep Springs Valley. Elemental variations are shown in a gradient of colors that varies from red for high abundance to blue for low abundance. Note the heterogeneous distribution of elements across the 2 × 2 mm surface domain sampled, a feature that is characteristic of the ten varnished clasts analyzed on the two terraces.

The Z-300 GeoChem Pro mode was used to generate 2D relative concentration maps, commonly described as “heat maps”, across the 16 × 16 grid raster patterns from our in situ LIBS analysis at the five field sites. Two such elemental distribution maps for upper terrace sample T5 (37°19′1.416″ N, 118°5′25.698″ W; Munsell color = 5 YR 3/1, very dark grey) and lower terrace sample T13 (37.31656 N, 118.089933 W; Munsell color = 10 YR 3/1, very dark grey) at the Deep Springs Valley location are shown in Figure 9.

The panels for samples 5T and 13T in Figure 9 display the spatial distributions of elemental concentration variation across a 2 mm domain on the surface of the varnish layer for 10 elements—Mn, Fe, Si, Al, Na, Ba, Ca, Li, K and Ti. Elemental abundances display an uneven distribution across the surface of the varnish layer at the pixel scale of the heat maps. The distribution of Mn for the varnished clast on the lower terrace is fairly even, whereas it is concentrated in just half of the sampling domain for the clast on the upper terrace. The surface of the clast on the lower terrace is richer in Mn, Ba and Ca overall than that on the lower terrace, which displays comparatively elevated abundances of Si, Al and Na. The most striking feature of these maps is the complementary character of the elemental distributions; Ba and Ca distributions are spatially coherent with Mn, whereas Al and Na track Si distributions, Ti distributions track those for Fe, and Fe and Mn distributions are uncorrelated. Concentrations of Li and K are significant and fairly evenly distributed in both samples.

#### 5.3.2. Depth Profiling

Depth profiling is one of the unique capabilities of handheld LIBS. A single laser shot by LIBS provides an analysis of the sample surface, but tunneling in the sample at a single location is also possible as successive laser shots ablate the sample to progressively greater depths. As noted above, the varnished quartzite clast from Death Valley was analyzed in the laboratory prior to a depth profiling study prior to our use of another analyzer in the field.

Previous profiling studies undertaken initially using electron microprobe analysis and, more recently, energy dispersive X-ray analysis, provided important insights into the chemical characteristics of rock varnish and its formation. The internal texture of the varnish layer is characteristically defined by cyclic lamination at the micron scale of interspersed oxyhydroxide and aluminosilicate components, with the former rich in Mn, Ba and Fe but deficient in Si and Al [8,18,19,28,29,33,34,38,43,76]. The emission intensities of Mn in the varnish layer typically diminish with depth and, at the micron scale, Mn and Si display antithetic variation, whereas Ba and Ca display a positive covariation with Mn, as does Al with Si. Interrogation of spots of ~1 μm size on prehistoric flint from the Negev Desert of Israel [8] recorded a strong correlation between Mn and Ba but no covariation of Si, Al and Fe with Mn. Their depth profiling at ~1 μm intervals along a vertical transect into the varnish layer observed maximum Mn concentrations at ~5 μm depth, indicating the transfer of Mn from the surface of the varnish layer into its interior. Of particular relevance to this study is previous LIBS analysis of rock varnish [24,25,59]. These studies recorded the same primary common constituents—Mn, Fe, Al, Si, Mg, Ca and Ba, as well as Li and H, observing that emission intensities decreased sharply from the surface into the varnish layer.

**Figure 10:**
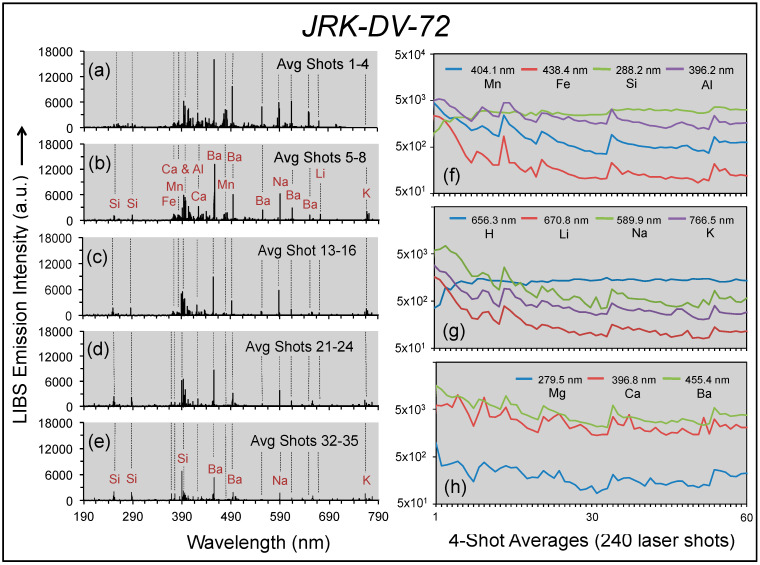
Depth profiles into the varnish layer of sample JRK-DV-72: (**a**–**e**) LIBS spectra for four-shot averages at five depths: shots 1–4 (**a**), shots 5–8 (**b**), shots 13–16 (**c**), shots 21–24 (**d**) and shots 32–35 (**e**); (**f**–**h**) emission line intensities for the 240 laser shot depth profile presented as semi-log plots for the 60 four-shot averages for Mn at 404.1 nm, Fe at 438.4 nm, Si at 288.2 nm and Al at 288.2 nm (**f**); H at 656.6 nm and alkali elements Li at 670.8 nm, Na at 589.0 nm and K at 766.5 nm (**g**); alkali earth elements Mg at 279.6 nm, Ca at 396.8 nm and Ba at 455.4 nm (**h**).

The results of our LIBS depth profiling into the varnish layer of Death Valley sample JRK-DV-72 with 240 laser shots, averaged into four-shot groups, are presented in Figure 10. The group averages over five depth intervals for laser shots 1–4, 5–8, 13–16, 21–24 and 31–35 are shown in panels (a–e). Although peaks for other elements are present in these LIBS spectra, none have significant signal-to-noise intensity to rise more than 2–3× above background. The averaged elemental emission intensities for the 60 intervals sampled are shown for Mn, Fe, Si and Al in panel (f), for H and alkali elements Li, Na and K in panel (g) and alkali earth elements Mg, Ca and Ba in panel (h). These three plots illustrate the extent of compositional variation within the portion of the varnish layer analyzed.

There are two notable features of our depth profile dataset. First, emission intensities for Mn and Ba are highest near the varnish surface, indicating the strong enrichment of the varnish surface in these elements described by previous studies. In this dataset for a single point on the varnish layer, Mn, Fe, Li, Na, K, and Ba emission intensities decrease sharply with depth into the varnish layer, whereas Al, Ca, and Mg exhibit less stark intensity variations along the depth profile. Secondly, in marked contrast to this general trend, Si (panel f) and H (panel g) exhibit a strong initial increase in emission intensity and then a diminished progressive increase further into the varnish layer. This antithetic behavior of Si and H compared to other elements is coherent throughout the depth profile, with variations for these two elements independent of those for Mn (R^2^_Si_ = −0.44, R^2^_H_ = −0.80). The repetitive spikes in spectral intensity observed for all elements except Si and H along the depth profile in Figure 10f–h are suggestive of a repetitive compositional variation through the varnish layer that could be interpreted to reflect multiple depositional cycles. The coherent behavior of Si and H suggests that silica plays an important and independent role in the development of rock varnish through the slow dissolution of silica in the presence of moisture from surficial material accumulations and then its subsequent gelling, condensation, and case hardening to form the indurated varnish layer [23,41] that incorporates the Mn-rich domains generated by cyanobacterial concentration [16].

#### 5.3.3. Future Work

LIBS analysis, combined with chemometric data processing, has been demonstrated as an effective way for specimen provenance discrimination [77,78,79,80]. Thus, in addition to in-depth investigations of elemental variations in rock varnish through detailed microchemical mapping and depth profiling in the short term, our subsequent effort will focus on using advanced statistical analysis to determine if the rock varnish developed on the desert pavements of the Deep Springs Valley alluvial fans of different age can be discriminated based on differences in their LIBS broadband spectra. Since LIBS plasma emission intensities are proportional to elemental concentration in the material under analysis, we will attempt quantitative analysis of rock varnish. The development of meaningful calibration curves requires having closely matrix-matched standards; however, because of the variety of chemical and physical effects on plasma emission [53], this facet of our future work will be directed toward laser ablation analysis to produce a suitable set of calibration curves for elements of interest in rock varnish (e.g., Mn, Fe, Si, Al, Li, Na, K, Mg, Ca and Ba) using the Z-300 GeochemPro application.

## 6. Summary

Presently, laser-induced breakdown spectroscopy (LIBS) is one of the two techniques for chemical analysis outside the laboratory and has been demonstrated to be effective for rapid qualitative and quantitative analysis of a broad spectrum of minerals, rocks, sediments and soils [58]. There are several attributes of LIBS that make it an attractive tool for the chemical analysis of geological materials. LIBS analysis is rapid (<1 s) and requires minimal or no sample preparation. LIBS can be undertaken in the laboratory or outside under ambient environmental conditions for in situ analysis on site for simultaneous multielement analysis of geological media in any state—solid (rocks, minerals, soils), liquid (natural waters, brines, hydrothermal fluids) or gas (e.g., ambient atmosphere, geogenic emissions). As a tool for analysis at the ~10 micron spatial scale, LIBS can also be used for the analysis of individual particles and mineral grains, surface microscale chemical imaging and compositional depth profiling. The other technique, X-ray fluorescence spectrometry (XRF), which is the most widely used handheld technique for field analysis of geological samples, has performance advantages for the analysis of base metals and rare earth elements due to its lower limits of detection. XRF benefits from factory-supplied calibrations that perform well across a wide range of sample types and thus is typically easier to use for quantitative analysis in the field due to the LIBS requirement for calibration on matrix-matched standards. However, XRF cannot measure elements of the atomic number lower than Si (16), has a 1–2 min analytical time depending on the required precision and detection limits and is burdened by licensing requirements, special operating precautions and operator safety training because of its open beam X-ray source. Since LIBS does not emit ionizing radiation, there are no special considerations for its operation or transportation other than a locking system that prohibits the laser from firing unless in contact with a sample, a condition met by the current generation of commercial handheld LIBS analyzers.

Overall, LIBS is complementary to XRF for material analysis and may be considered more versatile than XRF for analysis in the field because of its ability to identify light elements, its capability for surface microscale mapping and compositional profiling to depth and its safety, simplicity and rapidity of analysis. LIBS is also a more suitable tool for the analysis of rock varnish because handheld XRF and LIBS have very different areas of interrogation; compared to the area with the diameter of ~100 microns and the depth of ~10 microns excavated by the LIBS ablation process, a typical XRF analyzer integrates the composition of a surface area with the diameter of ~5 mm or more to the depth of a few hundred microns [81], which can extend below the thickness of a typical rock varnish veneer.

We investigated the chemical composition of rock varnish using handheld LIBS to determine its utility for three applications: elemental detection and identification, microchemical mapping and subsurface compositional profiling. In-situ LIBS analysis of rock varnish and visually similar coatings on granite outcrops and boulders across the Mojave Desert climatic region recognized the most abundant elements in rock varnish—Mn, Fe, Si, Al, Na, K, Mg, Ca and Ba along with the primary spectral lines for H, Li, Ti, V, Sr and Rb. Rastering of the laser beam over a 16 × 16 grid across 2 × 2 mm surface domains for varnish on multiple clasts on desert pavements on two alluvial terraces in Deep Springs Valley produced 2D maps of relative elemental concentration in real time for 10 elements—Mn, Fe, Si, Al, Na, Ba, Ca, Li, K and Ti. These microscale maps show that Ba, Ca, Li and Mn distributions in the rock varnish surface are spatially coherent, whereas Al and Na track spatial Si distributions. Depth profiling was undertaken on a varnished clast from Death Valley in the laboratory through ablation at a single point over 60 laser pulses. All elements, except for Si and H, are most abundant in the surface domain of the varnish layer, with their emission intensities decreasing into the varnish layer. These signal fluctuations record compositional variations with depth that can be interpreted as multiple depositional cycles. The distinctly different and coherent behavior of Si and H abundances through the depth profile compared to other elements suggests that the slow dissolution of silica in the presence of moisture from surficial material accumulations and its subsequent gelling, condensation and case hardening is an important process in the formation of rock varnish.

Overall, our LIBS analysis of rock varnish in Death, Owens, and Deep Springs valleys of east-central California are compatible with previous analytical results obtained using other analytical techniques, thus demonstrating the utility of handheld LIBS instruments for use in the laboratory as in situ analysis in the field. Although the current effort has been limited to qualitative assessments, the proportionality of LIBS plasma emission intensities to elemental concentrations provides a route for future development of matrix-matched calibration for rock varnish and quantitative elemental analysis.

## Figures and Tables

**Table 1:** Geochemical characteristics of rock varnish from California and western Nevada determined from the ICP-MS analysis compared with the elements identified in rock varnish across the Mojave Desert by LIBS.

ICP-MS								LIBS
California & Nevada								Mojave Desert
Major Elements		Minor Elements				Rare Earth		Elements
(wt. %)		(ppm)				Elements (ppm)		Identified
Na	0.059–1.790	Be	13–202	Y	13–518	La	11–1089	H
Mg	1.098–14.353	B	3–5	Zr	29–501	Ce	50–2593	Li
Al	7.290–11.187	S	4–44	Nb	19–54	Pr	9–224	C
Si	16.300–23.900	Sc	23–45	Mo	4–15	Nd	9–355	O
K	0.730–7.194	V	84–470	Ag	0.4–2.2	Pm	25–56	Na
Ca	0.313–8.340	Cr	8–1822	Cd	3–68	Sm	4–172	Mg
Ti	0.305–1.420	Co	18–684	Sn	4–6	Eu	1–32	Al
Mn	0.080–14.124	Ni	40–523	Sb	1–5	Gd	7–164	Si
Fe	4.010–16.200	Cu	44–1098	Cs	1–33	Tb	1–22	K
Ba	0.048–1.281	Zn	76–819	W	4–8	Dy	1–124	Ca
		Ga	15–26	Tl	1–6	Ho	1–25	Ti
		As	7–135	Pb	24–4510	Er	2–71	V
		Se	1–19	Bi	1–57	Tm	0.3–9	Mn
		Rb	19–350	Th	6–281	Yb	2–58	Fe
		Sr	225–1391	U	2–92	Lu	0.3–8	Rb
								Sr
								Ba

**Table 2:** Predominant LIBS spectral lines for elements identified in rock varnish across the Mojave Desert.

Element	Wavelength (nm)	Element	Wavelength (nm)	Element	Wavelength (nm)	Element	Wavelength (nm)	Element	Wavelength (nm)
H	656.28	Al	396.15	Ti	399.87	Mn	404.14	Fe	382.04
Li	670.79	Si	212.14	V	252.85	Mn	475.40	Fe	438.35
C	247.86	Si	288.16	V	263.07	Mn	476.24	Ba	455.40
O	777.19	K	766.49	V	400.57	Mn	478.34	Ba	489.99
Na	589.00	K	769.90	V	431.48	Mn	482.35	Ba	493.41
Na	589.90	Ca	393.37	Mn	257.61	Fe	234.35	Ba	553.55
Mg	279.50	Ca	396.85	Mn	259.37	Fe	238.20	Ba	614.17
Mg	518.36	Ca	422.67	Mn	260.57	Fe	239.56	Ba	659.53
Al	257.51	Ca	445.48	Mn	403.08	Fe	273.96	Sr	407.78
Al	309.27	Ti	334.94	Mn	403.31	Fe	274.93	Sr	460.73
Al	394.40	Ti	394.86	Mn	403.45	Fe	275.57	Rb	794.76

## Data Availability

The LIBS spectra for all of the samples described in this paper are archived in the LIBS Rock Varnish Analysis folder in the NCSU’s Data Management Plan Center (https://www.lib.ncsu.edu/do/data-management) (accessed on 23 August 2021).
